# The H19 Long non-coding RNA in cancer initiation, progression and metastasis – a proposed unifying theory

**DOI:** 10.1186/s12943-015-0458-2

**Published:** 2015-11-04

**Authors:** Eli Raveh, Imad J. Matouk, Michal Gilon, Abraham Hochberg

**Affiliations:** The Department of Biological Chemistry, The Alexander Silberman Institute of Life Sciences, Faculty of Sciences, The Edmond J. Safra Campus, The Hebrew University of Jerusalem, Jerusalem, 91904 Israel

**Keywords:** H19, miR-675, Tumorigenesis, Proliferation, Differentiation, EMT, MET, Genomic instability, miR-200, let-7

## Abstract

The imprinted oncofetal long non-coding RNA (lncRNA) H19 is expressed in the embryo, down-regulated at birth and then reappears in tumors. Its role in tumor initiation and progression has long been a subject of controversy, although accumulating data suggest that H19 is one of the major genes in cancer. It is actively involved in all stages of tumorigenesis and is expressed in almost every human cancer. In this review we delineate the various functions of H19 during the different stages in the complex process of tumor progression. H19 up-regulation allows cells to enter a “selfish” survival mode in response to stress conditions, such as destabilization of the genome and hypoxia, by accelerating their proliferation rate and increasing overall cellular resistance to stress. This response is tightly correlated with nullification, dysfunction or significant down-regulation of the master tumor suppressor gene P53. The growing evidence of H19’s involvement in both proliferation and differentiation processes, together with its involvement in epithelial to mesenchymal transition (EMT) and also mesenchymal to epithelial transition (MET), has led us to conclude that some of the recent disputes and discrepancies arising from current research findings can be resolved from a viewpoint supporting the oncogenic properties of H19. According to a holistic approach, the versatile, seemingly contradictory functions of H19 are essential to, and differentially harnessed by, the tumor cell depending on its context within the process of tumor progression.

## Background

Tumorigenesis is a multistep process that involves both the neoplastic tissue and its surroundings. In order to survive and flourish, cancer cells acquire a unique genetic background, proliferate rapidly, evade growth suppressors, cell death pathways and immune system attacks, and resist multiple drug treatments. Cancer cells are experts in managing hypoxia and stress conditions by recruiting blood supply to the neoplastic tissue, adjusting metabolically and adopting the plasticity that should enable epithelial to mesenchymal transition (EMT), metastasis and colonization at secondary sites [1]. In many aspects, a cancer cell resembles an embryonic cell: they share extraordinary plasticity, proliferation, motility and invasiveness capabilities, as well as the ability to make metabolic adjustments and other attributes, all orchestrated by common molecular pathways and epigenetic patterns [2].

One of the pivotal players in both embryonic development and tumorigenesis is the oncofetal lncRNA gene H19. H19, a maternally expressed and paternally imprinted 2.7 kb gene, resides close to the telomeric region of chromosome 11p15.5 and is reciprocally imprinted and regulated with its neighboring gene IGF2. Its genomic locus is rich in transcript coding sequences residing on both strands, as we have discussed elsewhere [3]. However, one of the most important transcripts in the H19 locus is miR-675, a highly conserved micro-RNA that resides within exon-1 of the H19 gene.

Recent studies have highlighted the important roles of H19 during the complex process of tumorigenesis, starting from the early stages that involve translational deregulation and genomic instability, through proliferation imbalance and stress management to metastasis. In this article we review the role of H19 in the tumorigenic multistep process and show how H19 touches almost every aspect of tumorigenesis. We also describe how H19 functions as an initial tumorigenic output component in the mammalian feedback system responding to various stress conditions. We suggest a general unifying theory that may answer some of the disputes and discrepancies that have arisen in regard to the fundamental characteristics of the *H19* gene.

## H19 mechanism of action

H19 has a highly conserved secondary structure. This evolutionary conservation suggests that H19's function is structure-dependent [4]. H19's function can be dissected into two major functions; a reservoir of miR-675 that suppresses its targets [5, 6], and a modulator of micro-RNAs or proteins via their binding.

MiR-675 targets a myriad of transcripts in a cellular-context-dependent manner [5]. For example, miR-675 directly downregulates Igf1r [5], Smad1, Smad5, and Cdc6 [7],Cadherin-11 [8], Cadherin-13 [9], Rb [10], Runx1 [11], Nodal Modulator 1 [12], TGFBI [13] CALN1 [14], and MITF [15]. Indirect targets of miR-675 were also reported and some of them will be mentioned below.

Increasing data suggests a role for the full length H19 transcript as a decoy for micro-RNAs that modulates their availability and suppresses their activity [16–18]. H19 was also found to interact with transcription-repressors and guide them to specific loci; H19 binds the methyl-CpG–binding domain protein 1 MBD1 and recruits it to some of its targets (including *H19*’s reciprocally imprinted gene *Igf2*), thus enables the maintaining of repressive H3K9me3 histone marks in their loci [19]. H19 also interacts with enhancer of zeste homolog 2 (EZH2), a histone H3 Lys 27 (H3K27) methyltransferase, that represses gene expression as part of the Polycomb-Repressive Complex 2 (PRC2) [20]. In fibroblasts,  H19 RNA itself is intracellularly sublocalized in lamellipodia and perinuclear regions through the binding of its 3’ to PTB (polypyrimidine tract-binding protein) and to 4 molecules of IGF2 mRNA-binding protein 1 (IGF2BP1) [21]. In gastric cancer, H19 was also found to bind P53 and partially inactivate it [22], and to bind ISM1 (Isthmin 1) in what seems to support a higher expression of this protein [14]. H19 was also found to bind the RNA binding protein K homology-type splicing regulatory protein (KSRP) [23]. This interaction supports KSRP targeting of unstable mRNAs, such as Myogenin, that leads to their subsequent decay. By that, H19 maintains an undifferentiated cellular state.

## H19 and P53: A tight relationship

Since *p53* is the major tumor suppressor gene in cancer, it is not surprising that *H19* and *p53* are mutually counter-regulated. Not only does P53 repress the promoter activity of the H19 gene [24, 25], it also epigenetically suppresses *H19* expression *in vivo* by inducing DNA demethylation of the imprinting control region (ICR) upstream to the *H19* gene [26]. We also have shown that hypoxia triggers *H19* expression in *p53* deficient cell lines [27], as will be later discussed. Moreover, it was recently found in bladder cancer cells that H19 derived miR-675 has a major role in inhibiting p53 and p53-dependent protein expression [28]. On the other hand, in gastric cancer cells H19 RNA was shown to interact with P53 protein, causing its partial inactivation [22], in what seems to be a negative feedback loop. As will be discussed below, this P53-H19 interplay is fundamental to understanding the role of H19 in tumorigenesis.

## H19 and genomic instability

Chromosomal instability (CIS) and overall genomic instability (including chromosomal aberrations and other mutations) were recognized as early as the beginning of the 20^th^ century by Bovery as one of the most basic tumor-enabling attributes of cancer (reviewed in [29, 30]). In the somatic cell, chromosomal stability and a balanced number of paired chromosomes are essential for appropriate gene expression and chromosome segregation during the normal cell cycle. Thus, the cell cycle process is kept under strict molecular surveillance to prevent unbalanced segregation, replication of damaged DNA and incomplete replication [30]. However, when vital caretaker genes (“genome guardians”) and gatekeeper genes (“proliferation buffers”) such as *p53, Rb* and others are dysfunctional, as in tumor initiation, the way is wide open for the accumulation of mutations and chromosomal missegregations [31]. These not only make the cell susceptible to future damage but also burden it with immediate metabolic stress, forcing the cell to accommodate to the alterations in its genomic content. On the other hand, it is these very genomic alterations that may confer the cell the genetic diversity necessary to manage stress [32, 33]. This possible advantage may provide an explanation for the various effects of hypoxic stress on genetic instability [34]; for the common polyploidy found in liver cells, which are under continuous oxidative and cytotoxic stress or following hepatectomy; and for other mechanical and metabolic stress conditions in which polyploidy is commonly reported (reviewed in [35]). As a comprehensive theory to explain the high rate of aneuploidy in tumors [36, 37], it was suggested that polyploidy is an intermediate, but sometimes unstable stage, that tends to attenuate proliferation (reviewed in [38, 39]) but also serves as a gateway karyotype to aneuploidy [30, 35, 40]. Moreover, the low proliferation rate of polyploid cells confers on them resistance to drugs that target actively cycling cells. Polyploid cells, due to their multiple but diverse chromosomes, have an inherent genetic system that buffers against deleterious mutations. Polyploidy cells also adopt attributes of stem cells, namely plasticity and metabolic reprogramming (reviewed in [40]), which make them an excellent starting point for more aggressive tumor descendants.

Recent studies by Zipori and colleagues [41, 42] have shown that H19 regulates polyploidy and that H19 expression is positively correlated with polyploidy suppression and tumorigenesis. These studies revealed that polyploid bone marrow mesenchymal stromal cells (BM MSC) have far less tumorigenic potential than diploid ones, have higher resistance to UV irradiation, and differ from diploid cells by their significantly lower *H19* expression levels. This correlation between *H19* expression and ploidy level was further demonstrated in mouse liver cells, which are diploid in the suckling mouse but drifted toward polyploidy with age progression while significantly decreasing their *H19* expression. Moreover, H19 knock-down in diploid MSC caused them to drift toward polyploidy and reduced their tumorigenic attributes and UV sensitivity. H19 knock-down also improved the efficiency of artificial tetraploidization in these cells. On the other hand, artificial tetraploidization itself reduced H19 levels, suggesting a negative feedback loop mechanism in which H19 represses polyploidy, which in turn represses H19 expression to maintain a polyploid state.

However, comparison of BM MSC with adipose MSC revealed lower *H19* expression in the latter cells, accompanied by diploid nature and a higher P53 level [42]. As both of the cell types had WT *p53* gene, the differences were probably due to epigenetic regulation of *p53* [42]. Indeed, when challenged with UV radiation, oxidative stress and chemotherapy, BM MSC responded by elevated expression of P53’s target genes, although the response was greater than in adipose MSC due to the relative low basal activity of p53 in BM MSC. This study provides further support for P53-H19 counter-regulation. Surprisingly, H19 levels were also reported to increase in tetraploid MSC in response to UV radiation [41].

**Fig. 1:**
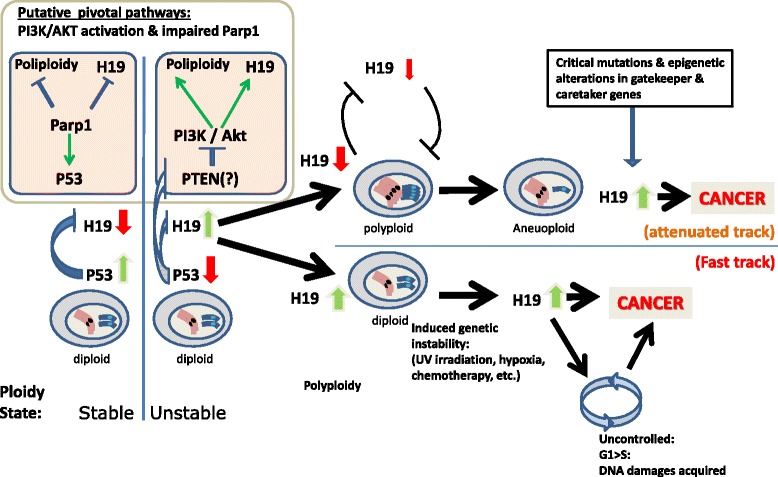
H19 in genomic instability. Cells maintain their normal ploidy state depending on normal expression of P53, possibly in a Parp-1 dependent manner. Reduction of P53 level (when Parp-1 is impaired?), leads either to polyploidy (attenuated track that prevents cancer progression) or to H19 upregulation (fast track to cancer, lower panel). PTEN > PI3K/Akt may be the basis of both routes. Since polyploidy (upper panel) is a common gateway to chromosomal loss/aneuploidy, which is usually accompanied with deleterious, tumorigenic mutations, it may eventually re-elicit H19 expression with its oncogenic properties

According to a model suggested by Zipori and colleagues based on their findings, P53, a pivotal determinant of CIS and a well-known genome keeper, interplays with H19 in cellular homeostasis. Upon downregulation of *p53*, cells are subjected to stress and can handle this by initiating either a selfish “cancerous plan” which is characterized by upregulated *H19*, or a “polyploidy plan” that enables the stressed cell to attenuate proliferation and acquire a genetic buffer from its high genomic content (Fig. 1). In the latter scenario, the cells enter a risky, temporarily stable state, which commonly leads later to aneuploidy as mentioned above. Lower levels of P53 together with the relatively unstable state of the polyploidy cell population may encourage a tumorigenic process in this population. The fact that H19 levels are increased in tetraploid MSC following UV radiation may support their pro-tumorigenic state. Aneuploid cells arising from the process described above may thus re-express *H19* at even a higher magnitude than that of their ancestor diploid population in order to further support tumorigenic attributes. Indeed, *H19* upregulation was reported in HeLa-skin fibroblast cell hybrids that became tumorigenic following the loss of one copy of chromosome 11 [43].

Despite the above, *H19* transient overexpression in diploid cells will not necessarily prevent polyploidy. In P53 WT hepatocellular carcinoma (HCC) cells, miR-675 transient overexpression actually increased the rate of tetraploid cells in culture [44]. However H19 and its derived miR-675 can down-regulate P53 activity, and this temporary P53-deficiency-related stress may be the cause of polyploidy, in accordance with the mechanism suggested above.

The mutually exclusive nature of polyploidy and H19 expression may also be inferred by the various reported effects of the natural drug curcumin. Curcumin treatment of various tumor cell types resulted in H19 suppression [45]. As curcumin induces mitotic abnormalities and subsequent cytokinesis failure that are usually the basis of polyploidy [46], the suppression of H19 by curcumin aligns with the genome destabilization effects of curcumin.

It is worth mentioning here, however, that the mechanism by which H19 represses polyploidy is still unclear. The PI3K/Akt signaling pathway was recently proposed as a good candidate to connect the alternate cellular fates of high H19 or polyploidy [47]. We have previously shown that PI3K/Akt active signaling upregulates H19 to promote metastasis [48]. Apparently, PI3K/Akt is also critical for the polyploidization phenomenon in the murine liver during weaning progression [49]. It is therefore tempting to assume that the PI3K pathway is upstream to the two alternative pathways that lead the stressed cells in opposite directions. This suggestion is further strengthened by the fact that P53 negatively regulates the PI3K pathway via transcriptional activation of its pivotal negative regulator, PTEN (which is also a negative regulator of H19 and miR-675 [50]). Moreover, P53 can also repress PI3K downstream effectors (reviewed in [51]).

Another candidate upstream of H19/Polyploidy fates fork is PARP-1 (poly ADP-ribose polymerase). PARP-1, a DNA damage repair enzyme and a positive regulator and stabilizer of P53 [52], was reported as a repressor of *H19* expression in ES cells [53]. But PARP-1 also prevents polyploidy as was demonstrated in PARP-1 null fibroblasts [54]. Therefore, it is also possible that impaired PARP-1, which is combined with, or resulting in, P53 reduction, accounts for either *H19* upregulation or polyploidization.

## H19 and P53 at the junction of the hypoxic stress response and proliferation

One of the fundamental hallmarks of cancer is uncontrolled, chronic cell proliferation. However, it is not only an attribute of cancer but also one of the major engines that drive carcinogenesis [55, 56]. The higher the rate of proliferation, the greater the risk of acquiring either antimorphic mutations (resulting in oncogenic activity) or amorphic mutations (in tumor suppressor genes (TSGs)) that may further promote proliferation [55]. Two major regulators of cell proliferation and TSGs, P53 and RB, are strongly affected by H19, which counteracts their action. As mentioned above, P53 is an H19 suppressor.

Hypoxia is a common stress condition in tumors. In addition to the abnormal vascularization commonly affecting their essential nutrient and gas exchange [57], the high proliferation rate of transformed cells impedes their access to normal blood supply. Thus, it is not surprising that hypoxia is a characteristic of all solid tumors [58]. Hypoxia, especially of the acute type, also tends to induce a more aggressive, invasive and resistant tumor phenotype [59, 60].

We have previously shown that hypoxia or artificial-hypoxia conditions induce *H19* transcription [61] when P53 is null or impaired [27]. Hence, under *in vivo* conditions, p53 mutations which are pro-proliferative can easily lead to tissue hypoxic foci in which *H19* expression is subsequently induced. As we have demonstrated [27], HIF1-α mediates H19 induction upon hypoxia when p53 is impaired, possibly due to the loss of P53 inhibitory effect on HIF1-α. Apparently, nuclear localization of P53, rather than its tetramerization (which is essential for the translational activity of P53 protein), is essential for H19 inhibition [27].

It has been shown in both liver and bladder cancer cells, both *in vitro* and *in vivo*, that upon induction, H19 supports tumor growth by suppressing the cyclin-dependent kinase inhibitor p57^kip2^ (CDKN1C) and other putative tumor suppressor genes, while upregulating pro-oncogenic genes (like cyclin E2 and others) [61]. H19 suppression of p57^kip2^ was evident also upon serum depletion, a condition that usually induces p57^kip2^ mediated quiescence [62]. Hence, once induced, H19 may worsen the hypoxic stress, initiating a positive feedback loop that further promotes proliferation and subsequent hypoxia. At the same time, as evident from our differential transcriptome analysis studies conducted both in liver and bladder cancer cell lines [61, 62], H19 enhances tumor survival under harsh conditions. H19 upregulates transcription of angiogenic genes and thus enables blood supply to the proliferating tumor. H19 also suppresses apoptotic-signaling-related gene transcription (among them DNA-damage sensitive genes, like the *DDIT3* gene), and promotes transcription of survival-related and chemo-resistance related genes [61, 62]. By resisting apoptosis under hypoxia, which is in itself a mutagenesis supporting condition [34], H19 allows further accumulation of deleterious mutations. Cells under acute hypoxia are prone to experience subsequent oxidative stress, caused by rapid hypoxia/anoxia and reoxygenation transitions, due to the abnormal vasculature in the tumor niche [63]. Intriguingly, it was reported that H19 post-transcriptionally upregulates Thioredoxin (Trx) protein in cancerous epithelial mammary cells [64]. Trx, a component in the system controlling the reduced intracellular redox environment and an abundant anti-apoptotic protein in many cancers, provides defense against oxidative stress [65]. Thus, it is possible that H19 also confers resistance to oxidative stress on the cancerous tissue. Indeed, H19 increased the promoter activity of the survival factor *NF-KB*, a validated target of Trx, as shown in luciferase assays [64].

Interestingly, another unexplored possible connection between oxidative stress and H19 promoted proliferation may be suggested from its proliferative effect in bladder cancer cells that was reported to be exerted by upregulation of *Id2* (inhibitor of DNA binding/differentiation 2) [66]. It was reported elsewhere that the levels of glutathione, a physiological antioxidant, are positively correlated with ID2 levels [67]. Hence, it seems that *Id2’s* sensitivity to hypoxic stress may be circumvented by H19 which may upregulate *Id2* regardless of hypoxic status.

In order to promote G1/S transition during the cell cycle, H19 needs to avoid suppression by P53 but also by Rb, another classic suppressor of G1/S transition and subsequent proliferation. One of the key proteins suppressed by Rb is E2F1, a transcription factor that binds to and activates *H19* promoter, as was shown in breast cancer cells [68]. Since E2F1 is known as a G1/S transition promoting factor during the cell cycle, it is reasonable to assume that H19 mediates the pro-proliferative function of E2F1 [68]. Indeed, silencing of H19 in breast cancer cells reduces their proliferation while *H19* overexpression accelerates cell cycle progression [68, 69]. Moreover, it was experimentally shown in breast cancer cells that Rb indirectly suppresses *H19* expression by repressing E2F1 [68], while in colorectal cancer cells [10] and in hepatocellular carcinoma cells [44], H19 derived miR-675 negatively regulates Rb expression. This phenomenon was verified in a transgenic mouse model of prostate cancer (*Pten* and *Zbtb7a* double knockout mice), in which Sox9 is upregulated and suppresses Rb via miR-675 [50]. The above data, when integrated, suggest a feedback loop in which upregulated H19 further promotes proliferation by repressing Rb [70]. This loop may be accelerated in response to stress, since H19 is upregulated following release from quiescence that was induced by serum starvation, and E2F1 is a known major player in quiescence to proliferation transition [68]. Hence, H19 responds not only to hypoxic stress but also to serum-depletion-induced quiescence by accelerating proliferation, dependent on the loss or dysfunction of critical TSGs.

**Fig. 2:**
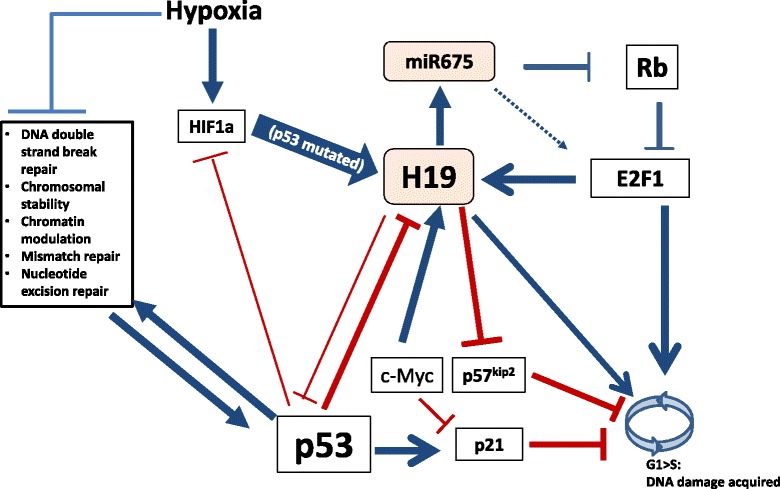
H19 in proliferation and in response to hypoxic stress. Hypoxia induces H19 expression under hypoxic stress in p53 mutated cells, in which HIF1α, which is essential to H19 upregulation, as well as H19 itself, are not repressed by P53. Since hypoxia is a mutagenic condition *per se*, it can also induce p53 mutations and cause the H19 response. H19 promotes cell cycle progression through Rb suppression by its miR-675 or by p57^kip2^ (CDKN1C) suppression or c-Myc activation

The convergence of P53 and Rb pathways to the H19 pivot (Fig. 2), together with the negative feedback exerted by H19 on both of those two master TSGs, may explain the gradual deterioration towards cancer, given the loss of only one of the TSGs, in spite of the apparent redundancy of the two pathways. Examples of this redundancy are discussed in ref. [1].

## H19 in epithelial to mesenchymal transition (EMT) and its converse MET process: a matter of circumstances?

Metastases are the major cause of cancer related death. Although proliferation and metastasis are uncoupled processes, it is plausible, at least according to the linear progression paradigm that suggests an evolutionary progress towards metstasis, that hyper-proliferation increases the somatic mutational rate that may promote a metastatic transition [1, 71, 72]. But regardless of its involvement in proliferation that may indirectly promote malignancy, recent studies by us and others show that H19 promotes tumor metastasis by direct involvement in malignant processes. Metastasis is a multi-step process consisting of apparently contrary sub-processes. EMT requires de-differentiation of epithelial cells in order to enable dissemination, migration through the extra cellular matrix (ECM) and intravasation into blood vessels that will carry them to their secondary site. Once extravased to the secondary site, the tumor cell goes through the reverse mechanism of mesenchymal to epithelial transition (MET) that resembles re-differentiation, and then (even decades later, in some cancers) proliferates and colonizes to become a secondary tumor [72]. Controversial reports describe H19 as either a pro-differentiation or a pro-proliferation factor; either an EMT promoter or an MET promoter. We will discuss these discrepancies in the context of metastasis as a whole.

## H19 in EMT

We have recently shown that numerous bona-fide EMT inducers also induce H19/miR-675 expression [48]. In HCC cells, TGF-β has been shown to induce H19 expression via activation of the PI3K/Akt signaling pathway. This TGF-β dependent induction of H19 has been recently corroborated in an experimental model of colorectal cancer [18]. We have also found that H19 is essential for upregulation of the EMT related transcription factor Slug by TGF-β. In addition, hypoxia induces H19 and miR-675 as well as the EMT markers Slug and Snail in breast cancer cells. Association of drug resistance with elevated levels of H19 and Slug was demonstrated in a cisplatin resistant ovarian cancer cell line, in which H19 upregulates, apparently indirectly, the promoter activity of Slug, which upregulates H19 in a positive feedback loop. We have also shown that in a pancreatic cancer cell line E-cadherin is totally ablated upon *H19* expression as long as H19’s miR-675 seed region is intact. This total ablation of E-cadherin was also shown in HCC and lung carcinoma cells overexpressing H19, together with the upregulation of the mesenchymal N-cadherin [48]. Besides enabling dissemination, E-cadherin loss is an essential step towards enabling cells to adopt their cytoskeleton to migrational mode [73]. Indeed, our studies suggest that H19 participates in the invasion process of cancer cells; Forced expression of H19 improves HCC cell invasiveness in an *in vitro* assay. Hepatocyte growth factor/scatter factor (HGF/SF), a ligand of the tyrosine kinase c-Met receptor named after its pro-migratory attributes, enhances H19 promoter activity in treated cells in culture. Moreover, anchorage independent growth assays suggested that H19 is essential for HGF/SF induced scattering morphology and colony formation in soft agar [48].

There is a large amount of *in vivo* evidence supporting the pro-metastatic nature of H19. Sub-colonies of a mammary gland tumor cell line differ not only in their ability to migrate to the lung and colonialize there, but also by their relative *H19* expression levels [48, 74]. In an experimental mouse model of lung carcinoma metastasis that was induced by intravenous injection of cells, cells overexpressing *H19* manifested improved ability to metastasize in comparison to control cells transfected with empty vector. Examination of lungs for micrometastases (that may serve as a measure of ability to metastasize) and macrometastes (visible metastases; a possible measure of proliferation) revealed that overexpression of *H19* resulted in an increased number of lung micrometastases compared to control mice. Macrometastes were only seen in mice injected with cells overexpressing *H19*, suggesting that the pro-proliferative attributes of H19 are also manifested at the secondary site [48]. In light of all the above, it is not surprising that high H19 levels are present in human biopsies of all common metastatic sites tested in our study, regardless of tumor primary origin [48].

Recently, and concurrently with our work, other researchers have found additional key players in the EMT process that are linked to H19, thus extending the network of routes through which H19 exerts its metastatic function. In bladder cancer cells, H19 was found both *in vitro* and *in vivo* to promote EMT, down-regulation of E-cadherin and cell migration. By association with enhancer of zeste homolog 2 (EZH2), a well-established mediator of tumor metastasis [75], H19 upregulates Wnt/β-catenin signaling, which in turn represses E-cadherin expression [20].

H19 can also promote metastasis through sponging and sequestering of the first identified micro-RNA, let-7 [16]. This let-7-sponging function of H19, which is conserved in both human and mouse, does not affect let-7 RNA levels but does affect its function as a suppressor of differentiation-suppressing-genes in muscle cells (such as Hmga2 and DICER). Hence, H19 may actually serve as a differentiation suppressor [16]. In accordance with its negative role in muscle differentiation, it was recently reported that H19 may act as a scaffold that favors KSRP-mediated degradation of myogenin transcript to subsequently contribute to the maintenance of the undifferentiated state of C2C12 muscle cells [23]. Given the differentiation suppressive attributes of H19 in muscle, it is not surprising that in pancreatic ductal adenocarcinoma, as well as in ovarian cancer and uterine serous carcinoma cell lines, sequestering of let-7 by H19 is essential for H19 function in EMT processes, including cell invasion and migration [76, 77]. In ovarian cancer and uterine serous carcinoma cell lines, the pro metastatic oncogene c-Myc [78, 79], which upregulates H19 [80], is also its indirect target, due to removal of c-Myc let-7-mediated repression. The anti-diabetic drug Metformin can vitiate the metastatic phenotype of ovarian cancer cell lines, apparently due to induction of hypermethylation at the H19 locus [77]. In this context it is highly intriguing to note that the H19/let-7 axis plays another role in muscle: while let-7 inhibits glucose uptake and promotes glucose intolerance, H19 upregulates insulin receptor in diabetic conditions to increase glucose uptake and is downregulated upon hyperinsulinemia [81]. Exactly how Metformin’s negative effect on H19 aligns with its function in increasing glucose uptake in skeletal muscle [82] (a function which is also attributed to H19) remains to be explored. Nevertheless, let-7 is not only sequestered by H19, but also can destabilize it under hyperinsulinemia conditions (as opposed to the highly stabile status of H19 in normal differentiated muscle [83]); therefore it exerts a negative feedback cycle to repress its negative regulator H19 [81].

A recent study contradicts the above findings since it reported pro-differentiation properties of H19 and its derived miR-675 forms in muscle [7]. Descriptive evidence indicates that miR-675 is upregulated gradually upon murine skeletal muscle injury *in vivo* to mediate muscle regeneration and differentiation through inhibition of the BMP-pathway components. This study however found that let-7 was not induced in their cell culture model during differentiation, even though the same muscle cell line was used. Thus, it is possible that H19 may be capable of totally opposite functions, with the specific functions being largely dependent on cellular context and partners. We will come back to this point below.

The most important lesson that can be learnt from the H19/let7 axis findings is that positive correlation between a specific factor and a phenotype may suggest the opposite to what one would assume. Instead of being a differentiation factor in muscle cells, as has been thought till recently, H19 is upregulated during myo-differentiation mainly in order to counteract the pro-differentiation functions of let-7 [16]. As H19 harbors not only let-7 binding sites (both canonical and non-canonical), but also other putative miRNA binding sites [16], it is plausible that other miRNA interplay with H19 in various ways. Indeed, it was recently reported that miR-106a is also sponged by H19 in both Hela cells and myoblasts [17]. Another fascinating finding suggests that H19 promotes EMT by sponging two EMT repressors, miR-134 and miR-200 in colorectal cancer metastasis [18]. It would seem that the finding of differentiation-correlated expression of H19 in human BM MSC [84], for example, should prompt us to search for possible H19 targets/partners such as let-7 or miR-106a.

Another characteristic of an EMT process is the stemness phenotype of the transformed cells. The stem cell-like state of cells during EMT enables them to self-renew and proliferate while transforming, disseminating and migrating [85]. Interestingly, *H19* maternal expression which directly impacts upregulation of Igf2 and Igf1r, together with the repressive effect of miR-675 on Igf1r [5], may take part in the maintenance of adult haematopoietic stem cell quiescence [86]. Furthermore, a recent study of prostate and breast human cell lines revealed a putative role of stemness for *H19*, since its expression was positively correlated with stem cells markers and pluripotency factors. H19 was also found to control the expression of two major pluripotency factors, Oct4 and Sox2, which apparently regulate H19 in an amplification feedback loop [87].

## H19 may act differentially depending on developmental stage

In spite of the strong supportive evidence for H19 and miR-675 involvement in EMT, which is regarded as a de-differentiation/trans-differentiation process, there is enough contradictory data to position H19 as a tumor suppressor and a pro-differentiation factor. However, when the data are carefully examined, it would seem that most of the controversy may be solved by dividing the functions of H19 into two developmental periods. We propose that in the embryonic period H19 mostly promotes differentiation, while in the adult it is rarely expressed in noncancerous tissues and has tumorigenic properties. Moreover, nullification of H19 or severely abrogating its function in cells at early developmental stages may impact its function at later stages, making the cells more liable to tumorigenesis. The significance of the embryonic expression of H19 for later stages is implied as H19 expression in embryonic samples controls the gene expression of several imprinted genes of the imprinted gene network (IGN) by facilitating deposition of repressive histone marks on their differentially methylated regions [88], mostly by recruiting MBD1 protein to their loci [19]. Regardless of the different nature of the genes H19 controls by this mechanism (for example: the cancer related Igf2 on one hand and cell cycle inhibitor Cdkn1c and Igf2r growth repressor on the other), the model used to show H19 control over the IGN was based on H19 KO (knockout) mice manifesting an overgrowth phenotype and subsequent mating of H19 transgenic with the KO mice to rescue the phenotype [88]. Assuming that the embryonic stage of H19 expression is a critical stage that impacts a set of other imprinted genes, it is not impossible that conditional silencing of the H19 in the adult would not have rescued the overgrowth phenotype, or that conditional expression of H19 in the adult would not have achieved the phenotypic rescue reported. According to our hypothesis, in the second scenario, induction of H19 at later stages may even worsen the tumorigenic phenotype. This suggested distinction between embryo and adult was previously implied by Gabory and colleagues, who studied the H19 KO mouse model described above [89].

In support of our suggestion, H19 expression leads to growth retardation, abrogation of clonogenicity and impaired *in vivo* tumorigenesis, but these findings were manifested in embryonic tumor cell lines [90]. Another comprehensive study in murine KO models isolated the H19 deletion effect from the effect of Igf2 overexpression, which commonly accompanies H19 deletion, and showed in several tumor models that H19 has tumor suppressing properties [91]. However the researchers that used this murine model also disregarded possible potential implications of lack of H19 expression during embryonic stages. Moreover, they used ES cells in one of their models. The researchers also admit that H19 KO mice do not tend to spontaneously develop tumors, which indicates that H19 is not a tumor suppressor *per se* but rather its deletion increases cells tendency for cancer. We suggest that this deletion is critical at the embryonic stage and can also impact later stages.

## The emerging role of H19 in MET

As we have seen above, H19 plays a role in EMT. Can it also play a role in MET? Is MET, in its literal meaning of re-differentiation, needed for metastasis at all where H19 participates? Metastasis can be explained as a result of either MET, the process of re-differentiation after intravasation of the tumor cell to its secondary site, or plasticity and stemness of the metastatic cells [85]. Plasticity does not require continuous genetic alterations, except for initiator mutations. Instead, cells adopt semi-stemness and primitive differentiation attributes that confer on them the ability to accommodate and react to environmental cues, while keeping their high proliferative potential. Plasticity may be supported by reversible epigenetic patterns but other mechanisms are also possible.

We previously mentioned that H19 is highly abundant in secondary tumors and in cell lines that complete their journey to the secondary site. We have reported that miR-675 is essential for EMT, ablation of epithelial markers and upregulation of mesenchymal markers as found in hepatocellular carcinoma *in vitro*. An apparently conflicting report indicated that miR-675, in another hepatocellular carcinoma cell line model, is an MET promoter [44]. While increasing proliferation and repressing Rb, miR-675 altered cellular morphology, upregulated epithelial markers and downregulated the mesenchymal ones, reduced invasive potential, and increased anchorage-independent growth capacity. In addition, it downregulated the EMT key mediator, Twist1 (as opposed to its high positive correlation with H19 in the murine metastasis model of 4 T1 breast cancer cell line in which Twist was initially identified as a metastasis marker [74]). In prostate cancer cells, miR-675 was also reported to suppress the extra cellular matrix TGF-β induced (TGFBI) protein transcript, which enhances motility and invasion in metastasis. [13]. Those findings also align with the study that claimed a pro-differentiation role for miR-675 in muscle, as mentioned above [7].

## “Selfish” H19 promotes metastasis along its seemingly contradicting stages

**Fig. 3:**
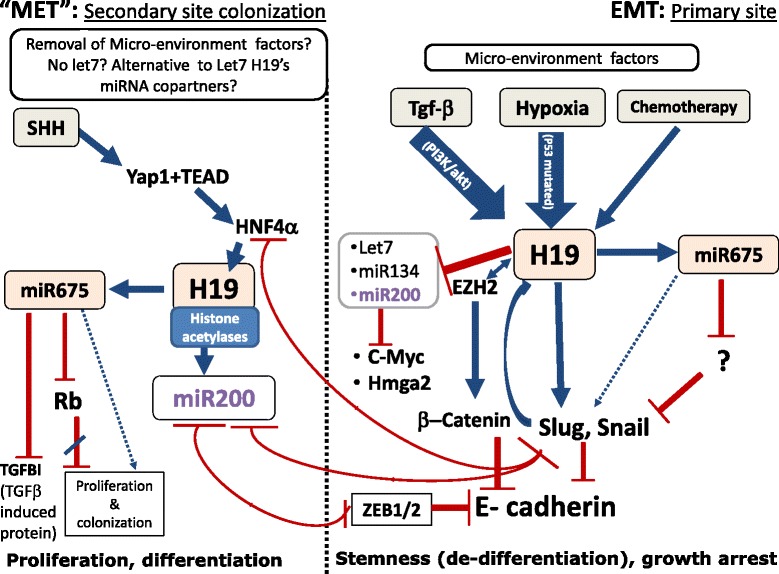
H19 in metastasis. H19 plays a role in both epithelial to mesenchymal transition in the primary tumor and colonization and re-differentiation/accommodation to niche in the secondary site. As delineated above, various contradictory pathways are controlled by H19 in the two scenarios. H19 is consolidated into the process active at that time, which is a function of the micro-environmental factors, and may find other partners in each of the scenarios (miR-200 and let-7 in EMT and putative alternative partners such as histone acetylases in MET)

We believe that the plasticity and stemness model we described above may resolve the problems raised by the contradictory functions attributed to H19 (Fig. 3). This model also takes into account the key players in two known pivotal negative feedback loops related to EMT-MET balance: the ZEB1 - miR-200 loop and the Snail – miR-34 loop [85]. ZEB1 and Snail induce EMT, growth arrest, drug resistance and stemness in response to TGF-β, hypoxia, chemotherapy and other inducers. In fact, H19 shares the same inducers and effectors of EMT as ZEB1 and Snail, as detailed above. However, ZEB1 and Snail are negatively counter-regulated at the transcriptional level by miR200 and miR34, respectively. These miRs are in turn downregulated by their target Snail [92]. MiR-200 and miR-34 drive MET, differentiation, proliferation and drug sensitivity. These feedback loops are a mechanistic solution that provides a balance between the two opposite processes. Interestingly, H19 was shown to suppress EMT through upregulation of miR-200 family members by interaction with protein complexes that induce histone acetylation upstream to several miR200 genes. Subsequently, miR-200 mediates H19 dependent down-regulation of Snail and Twist [93]. Furthermore, the murine 4 T1 cell line mentioned above, the only breast cancer cell line among four clones that completes a breast to lung metastasis, was also the only one to express high miR-200 and H19 levels [48, 74, 94].

Another fascinating regulatory level lies in the inhibitory circuit between Snail, the repressor of miR-34 and miR-200, and HNF4α, which, contrary to Snail, promotes differentiation [92, 95]. HNF4α is a transcription factor which is abundant in both hepatoblasts (the liver bipotential cells that during development progressively differentiate into hepatocytes and cholangiocytes), and in the differentiated hepatocytes. However, it turns out that the distinct functions of HNF4α at different developmental stages of liver cells, from embryo to adult, are due to the developmentally dependent binding pattern of HNF4α to differential enhancers. Intriguingly, one of the HNF4α enriched enhancers in embryonic liver belongs to the *H19* gene. Moreover, it was shown that the transcriptional co-regulator Yap1, which is the nuclear effector of the Hippo signaling pathway, induces the binding of HNF4α to *H19* enhancer and upregulates its expression in the embryonic liver via activation of the transcription factor TEAD, a classical cofactor of Yap1 [96]. Yap1 promotes metastasis via TEAD [97], and was recently found to upregulate H19 in osteosarcoma in response to Hedgehog signaling activation [98].

In light of the above, we suggest that H19 may support both EMT and MET (Fig. 3), conferring the cell the plasticity needed throughout the stages of transformation. H19 plays a role in EMT that eventually consolidates with that of ZEB1 in EMT, due to their shared extracellular EMT inducers. Moreover, EMT inducers such as TGF-β also have a negative impact on miR-200 expression [99, 100], an impact that is further supported by H19’s direct sponging of miR-200. H19 also induces Snail expression as we have previously reported. However, in regard to the ZEB1–miR200 loop, H19 rather promotes the MET axis, apparently by interaction with a ribonucleoprotein complex that activates miR200 expression [93]. This interaction may explain why in MET, H19 serves rather as an inducer of miR-200 than as its sponging repressor [18]. Hence, upon reduction or removal of EMT extracellular inducers like TGF-β, or activation of Hedgehog signaling (an established Yap1 activator [98, 101]) or, possibly, inactivation of hippo signaling (which represses Yap1 activation), H19 shifts the cell to an “MET” state. This shift may be further supported by upregulation of ribonucleoprotein complexes, such as histone acetylases [93, 102], that compete for H19 binding to exert an MET-like phenotype.

It is also possible that H19 expression is alternatively induced by the converse transcription factors Snail and HNF4α in each of the opposite scenarios of EMT and MET. These two factors may not only suppress each other but also compete on binding to the H19 enhancer, though this suggestion should be further explored.

Of note, P53 is a known inducer of both miR-200 and miR-34 and hence supports MET (revieved in [85]). Nevertheless, we have pointed out above that P53 and H19 are, in general, mutually regulated. It is therefore appealing to suggest that H19 is an alternative, P53 independent MET promoter, which is more favorable to continuous cancer progression than to metastasis arrest given its contribution to both MET and EMT.

## Other transcripts on the H19 locus

As we have reviewed extensively elsewhere [3], two major antisense transcripts reside on *H19’s* opposite strand; the 120 kb-long 91H transcript and the smaller ~6 kb HOTS transcript. The unstable 91H [103], as opposed to H19, is exclusively nuclear, however it shares some of H19 attributes. It is also induced during myoblasts differentiation and upregulated in cancer (due to RNA stabilization), as was shown in breast cancer cell lines. However its silencing, though it has negligible effect on *H19* expression, reduces the expression of its reciprocally imprinted gene, *Igf2,* in contrary to H19’s suppressive effect on Igf2 expression [104]. The HOTS transcript [105] encodes for nuclear protein in primates but lacks an open reading frame in mouse. It is still unclear whether it is an autonomous transcript or a part of 91H. The last option is less trivial, since, according to in vitro data, HOTS functions as a tumor-suppressor. Although loss of *IGF2* imprinting leads to silencing of both H19 and HOTS, the expression of *H19* and *HOTS* seems to be uncoordinated, as it is not mutually correlated across tissues. Moreover- expression *in vitro* of each does not affect significantly the expression of the other. Although the scanty data available as for the two *H19* antisense transcripts is not concrete enough to conclude what their effect on H19’s role in tumorigenesis might be, and in spite of the mutually independent regulation between them and H19, they may counteract H19 function. A possible balance between H19 and its antisense transcripts may serve as an additional regulatory level that may dictate cellular fate.

## Conclusion

**Fig. 4:**
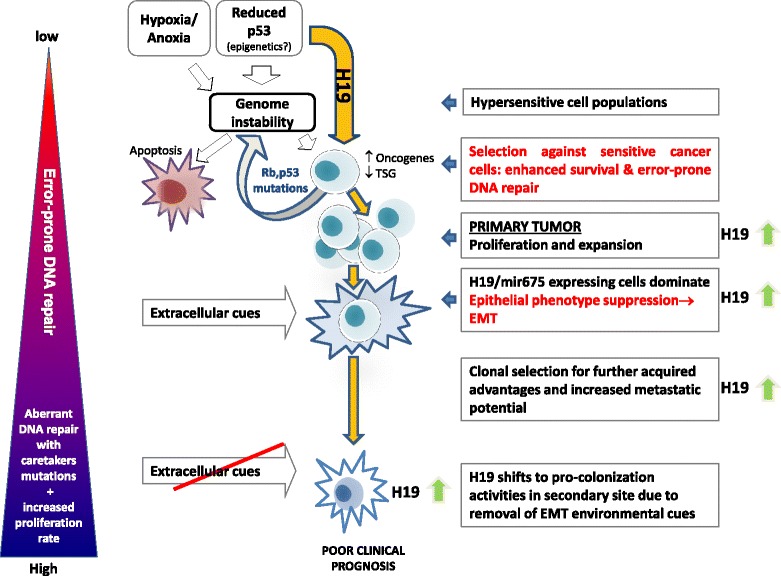
Outlines for H19 functions during tumor progression. Various stress conditions drive genomic instability in its wide meaning of mutation and chromosomal abnormalities. In some cases, such as severe reduction in P53 levels, H19 upregulation in sub-clones of stressed cells is a direct cancerous reaction to stress that drives proliferation and accelerates mutational rate. H19 acts to enable a selfish cellular survival plan and reacts to stress conditions by accelerating proliferation rate. H19 subsequently promotes the metastatic cascade from EMT in the primary tumor to metastasizing in secondary sites, depending on extracellular and intercellular context

This review has taken us on a journey along the main stations of tumorigenesis, spotlighting H19. We have found that H19 responds to various stress conditions such as reduced P53 and hypoxia, by activation of a tumorigenic, selfish program of cell survival. H19 opposes polyploidy-mediated growth arrest. Subsequent hyperproliferation naturally increases acquisition of further mutations in TSGs and oncogenes that unleash tumor progression. Following proliferation and expansion, hypoxia and other triggers induce EMT, cell invasion and extravasation. Due to possible extracellular signaling shift in the secondary site to which the tumor cell has intravasated, H19 may support MET, colonization and proliferation, processes in which H19 was already involved at the very initial stages of tumor formation at the primary site (illustrated in Fig. 4).

We have also highlighted the importance of H19 during embryonic stages, proposing that at embryonic stages H19 acts mostly as a differentiation promoter. We have proposed that those stages may be vital for shaping H19’s role and functions throughout development and may impact its seemingly opposite tumorigenic roles in the adult.

Some of the suggestions and hypotheses raised here should be further examined and explored deeply. Although we have used as holistic an approach as we could, we are aware of the differences between models and conditions that were used in the reports we reviewed. Indeed, to establish our proposed paradigm of H19’s involvement throughout tumor progression from its initiative events of translational deregulation and genomic instability to metastasis and secondary colonization, H19 should be studied in one, holistic, induced metastasis model throughout cancer progression. In addition, a correlation should be determined between the apparent functions of H19, which is accompanied by a phenotypic output in the certain cell line of interest, and the dynamic levels of the major players in the EMT–MET switch this cell line expresses. Nevertheless, there is no doubt that H19 is tightly linked to tumorigenesis throughout all its stages. In view of its deep involvement in cancer, H19 should be placed in the center of the combat against cancer as a main therapeutic target and a cancer marker.
